# Trastuzumab-Induced Interstitial Pneumonitis

**DOI:** 10.7759/cureus.42116

**Published:** 2023-07-19

**Authors:** Kimberly Errisuriz, Daniela Z Bazan, Rene Verduzco Jr., Rosa Guedez

**Affiliations:** 1 School of Pharmacy, Texas A&M Health Science Center, Kingsville, USA; 2 Pharmacy, Doctors Hospital at Renaissance, Edinburg, USA; 3 Internal Medicine, University of Texas Rio Grande Valley School of Medicine, Harlingen, USA

**Keywords:** docetaxel-trastuzumab-pertuzumab therapy, drug-induced pneumonitis, interstitial pneumonitis, trastuzumab-deruxtecan, trastuzumab emtansine

## Abstract

Trastuzumab is a recombinant immunoglobulin G1 monoclonal antibody used to treat human epidermal growth factor receptor 2 (HER2) cancers. Trastuzumab-induced interstitial pneumonitis is a rare adverse effect reported in a few patients. Interstitial pneumonitis presents as symptoms of dyspnea, hypoxia, cough, and fever. If the patient is treated early, corticosteroids can slow or reverse the disease progression.

A 41-year-old woman presented with dyspnea and a dry cough three weeks after her third cycle of trastuzumab therapy for breast cancer. A diagnosis of trastuzumab-induced interstitial pneumonitis was made after multiple other disease processes were ruled out. The patient was started on methylprednisolone while inpatient and transitioned to prednisone for outpatient therapy. The patient was maintained on 2-3L of oxygen throughout her hospital stay and was discharged on 3L of oxygen through nasal cannula. Trastuzumab was never restarted after discharge.

There have been many trials evaluating the safety, efficacy, and optimal treatment regimen of trastuzumab, but there are only a few reports of interstitial pneumonitis adverse reaction. The lack of correlation and limited cases make this adverse effect very difficult to diagnose and monitor. New trials and case reports can bring an insight into contributing factors, symptoms at onset, and treatment for future patients. With the increase in use of trastuzumab therapy, physicians should be aware of how to diagnose and treat the rare adverse reaction of trastuzumab-induced interstitial pneumonitis.

## Introduction

There is readily available guidance on how to treat interstitial lung disease (ILD) induced by human epidermal growth factor receptor 2 (HER2) therapies. However, there is limited information on how to diagnose and monitor ILD in patients [1,2]. We herein report a case of trastuzumab-induced interstitial pneumonitis.

This article was previously presented as a poster at the 2022 ASHP Midyear Conference on December 6, 2022, and at the 2022 ACCP Virtual Poster Symposium on May 25, 2022.

Drug-induced interstitial pneumonitis

ILD is a rare adverse effect of trastuzumab that has only been reported in a small number of cases. ILD consists of disorders of known and unknown etiology, with the latter referred to as idiopathic interstitial pneumonias [3]. ILD can occur when a patient is exposed to a drug that causes inflammation or fibrosis of the lung interstitium, which is further classified as drug-induced interstitial lung disease (DIILD) [3]. Common drugs that are associated with DIILD are cytotoxic agents, antibiotics, anti-rheumatic drugs, non-steroidal anti-inflammatory agents, psychiatric medications, and anti-arrhythmic agents [3]. Some risk factors for DIILD include age over 60 years, pre-existing lung disease, and a history of smoking [4]. DIILD is a diagnosis of exclusion, which is based on a patient’s history, physical examination, radiological findings, and laboratory data [4,5]. CT scans are the primary method of identification because they are non-invasive and provide the highest sensitivity and specificity in the detection of interstitial pneumonitis. The first week after lung injury, DIILD will manifest on the CT as diffuse ground-glass opacities and consolidations. After one to two weeks, it can present as irregular linear opacities, architectural distortion, and traction bronchiectasis [3]. Patients normally present symptoms of dyspnea, cough, fever, and hypoxia [6]. Corticosteroids are commonly used for ILD and can effectively slow or reverse the disease progression [1]. The mechanism of trastuzumab-associated lung injury is not clear; however, there are several studies with proposed theories [5]. One proposed theory is that type II pneumocytes are responsible for producing and secreting pulmonary surfactant, which creates low surface tension that prevents alveolar collapse [7]. HER2 is expressed by type II pneumocytes and is involved in cell proliferation and wound repair [8]. Trastuzumab is a HER2-targeted antibody drug that blocks HER2 from being expressed in the body [9]. Trastuzumab blocks HER2 from repairing any injuries to the lungs and secreting pulmonary surfactant, leading to an increased risk of acute lung injury [5,7].

Trastuzumab background information

HER2 is overexpressed in approximately 15-20% of metastatic breast cancers [10]. Trastuzumab is a recombinant immunoglobulin G1 monoclonal antibody that targets the extracellular domain of the HER2 protein [11,12]. Studies show that antibody-dependent cellular cytotoxicity (ADCC) is the major mechanism of action. Trastuzumab binds to the HER2 protein, and the Fc part of the drug is recognized and attacked by natural killer cells [13]. Patients with metastatic breast cancers treated with trastuzumab have had better prognosis and survival due to its inhibition of constitutive HER2 signaling and induction of ADCC [1,14]. Due to these better outcomes, the combination of an anti-HER2 antibody with taxane is now the established standard primary chemotherapy for metastatic breast cancers [12]. Trastuzumab is normally administered as an intravenous (IV) infusion once every 21 days [14]. Compared to other chemotherapies, trastuzumab is well tolerated and is not associated with alopecia, myelosuppression, and severe nausea and vomiting [15]. There are not many adverse effects associated with trastuzumab, but studies have shown rare occurrences of cardiotoxicity and interstitial pneumonitis [11].

## Case presentation

A 41-year-old woman presented to the emergency department (ED) from an oncology infusion center with complaints of shortness of breath prior to receiving her trastuzumab infusion. The patient had a past medical history of metastatic breast cancer (modified radical mastectomy 2017), essential hypertension, hyperlipidemia, hypothyroidism, and obesity. She had a worsening dry cough over the last three weeks associated with dyspnea. The patient denied having fever and chills. She reported having difficulty lying flat and had to sleep sitting up because of the shortness of breath. The patient had received her third cycle of trastuzumab therapy three weeks prior to presenting to the ED. Her oxygen saturation in the ED was documented at 84%, and the patient was tachycardic. The patient was admitted with a diagnosis of acute respiratory failure and pulmonary edema.

The patient reported drug allergies to ibuprofen that caused redness to the skin and acetaminophen with no documented reaction. Her home medications upon admission included atenolol, levothyroxine, losartan, and lovastatin.

**Figure 1:**
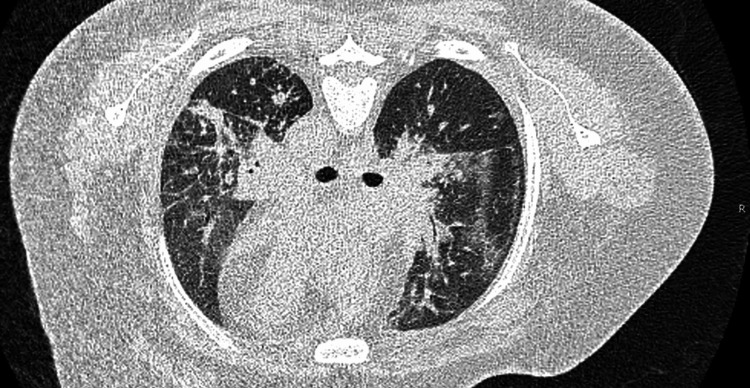
High-resolution CT of the chest without contrast High-resolution CT of the chest on day 4 indicating multifocal areas of airspace consolidation in each lung, most consistent with post-obstructive pneumonitis/atelectasis.

A respiratory viral panel was ordered on admission that included a SARS-CoV-2 PCR, which was negative. A chest X-ray obtained on admission indicated moderate to marked diffuse pulmonary edema. A CT angiogram (CTA) of the chest obtained on day 2 was negative for pulmonary embolism. The echocardiogram showed normal systolic and diastolic function, with no evidence of chemotherapy-related cardiomyopathy. A high-resolution CT of the chest obtained on day 4 indicated ground-glass opacities and thickening of the interlobar septae (Figure 1). Urine, blood, and respiratory cultures collected on admission from a bronchoalveolar lavage (BAL) all showed no growth of any organisms.

The patient was given a total furosemide dose of 60 mg IV in the ED for pulmonary edema. The patient was initially treated with enoxaparin 1 mg/kg subcutaneously for possible pulmonary embolism. Once CTA showed no signs of pulmonary emboli on day 2 (Table 1), enoxaparin dose was decreased to 40 mg subcutaneously daily for deep venous thrombosis prophylaxis. On admission, levofloxacin 750 mg IV every 24 hours and vancomycin 750 mg IV every 8 hours were started for possible community-acquired pneumonia. The patient's complete blood count was unremarkable except for an elevated white blood cell count of 11,000/uL. After 48 hours, the antibiotics were discontinued since the patient never experienced a fever, and the clinical picture did not fit with pneumonia.

**Table 1:** Patient’s radiology findings

Examination	Hospital day	Findings
Chest X-ray (one view)	Day 0	Moderate to marked diffuse pulmonary edema in a perihilar distribution
CT angiography of the chest	Day 2	No definitive central pulmonary emboli seen; Increased scattered bilateral areas of atelectasis as well as direct extension of the metastatic process into the lung parenchyma; scattered bilateral ground-glass opacities compatible with post-obstructive pneumonitis changes
High-resolution CT of the chest without contrast	Day 4	Multifocal areas of airspace consolidation in each lung, most consistent with post-obstructive pneumonitis/atelectasis; stable areas of ground-glass opacity and thickening of interlobar septae, could not exclude lymphantitic spread of carcinoma

The patient’s oxygen saturation at admission was 85% on room air and was started with 5L of oxygen through a nasal cannula. The oxygen improved to 97 on the nasal cannula and the flow rate was decreased to 3L. Oxygen flow rate remained between 2L and 3L throughout the rest of the stay, and the patient was discharged on 3L of oxygen through a nasal cannula.

A pulmonologist was consulted for this case, who agreed with the diagnosis of probable trastuzumab-induced pneumonitis with acute respiratory failure. Methylprednisolone 40 mg IV every 6 hours was started approximately 24 hours after presenting to the ED. The patient received 15 doses or approximately four days of this regimen. The patient was transitioned to prednisone 60 mg orally daily and completed a total of 7 days of corticosteroid treatment in the hospital. The patient was discharged with a prescription for prednisone 40 mg orally daily to continue for an additional week until she could follow up with the pulmonologist. The patient responded to steroid treatment. On day 5 of the hospital stay, she was still short of breath with minimal ambulation, but she was stable and ready for discharge with oxygen.

Breast cancer history

The patient was diagnosed with HER2-positive, and estrogen receptor, progesterone receptor (ERPR) negative breast cancer in 2016 and underwent a right modified radical mastectomy and axillary dissection in 2017. Upon initial diagnosis of breast cancer, the patient was started on a regimen of paclitaxel, trastuzumab, and pertuzumab. She then continued to receive trastuzumab and pertuzumab, followed by trastuzumab monotherapy for approximately two years. With the start of each regimen, the patient received 8 mg/kg loading dose of trastuzumab followed by 6 mg/kg maintenance dosing every three weeks. There were no serious adverse effects reported during this initial treatment period, and the patient did not receive any trastuzumab for almost two years.

The patient was then restarted on trastuzumab approximately two months prior to the admission. She received a loading dose of 8 mg/kg on her first cycle. She then received two additional cycles at three-week intervals of trastuzumab 6 mg/kg before presenting to the ED with complaints of shortness of breath. On this admission, the patient was diagnosed with trastuzumab-induced pneumonitis with acute respiratory failure and orders were placed to hold trastuzumab until the patient could follow up with her oncologist. Trastuzumab was never restarted after discharge.

At follow-up with her oncologist, a biopsy was taken that was indicative of lymphangitic spread of breast cancer. She was scheduled to begin another chemotherapy agent, 10 months after her trastuzumab-induced pneumonitis, but was oxygen-dependent at the time and continued to deteriorate. She was admitted to the hospital secondary to respiratory failure requiring intubation. After palliative care was consulted, she was extubated and passed away.

## Discussion

Trastuzumab is a widely used treatment for HER2-positive cancers since its FDA approval in 1998 [13]. There have been many trials evaluating its safety, efficacy, and optimal treatment regimen, but there are only a few reports of the interstitial pneumonitis adverse reaction (Tables 2, 3). In the phase III randomized HERA trial, trastuzumab was administered to 3,374 patients at a maintenance dose of 6 mg/kg, and there were no reports of interstitial pneumonitis [15]. In the National Surgical Adjuvant Breast and Bowel Project (NSABP) trial B-31, there were 1,015 patients treated with a regimen including trastuzumab, of whom four patients were reported to have interstitial pneumonitis [11]. The North Central Cancer Treatment Group trial N9831 had 809 patients treated with trastuzumab, of whom five patients had grade 3+ pneumonitis or pulmonary infiltrates [11]. Although trastuzumab is primarily used for breast cancer treatment, the adverse effect of DIILD was also reported in trastuzumab treatment for non-breast and non-gastric HER2 tumors [16]. The few case reports that have been reported (Table 2) do not indicate any correlation between the patients’ history, dosing regimen, or symptom onset. All of the patients reported (Table 2) were hospitalized and treated with a corticosteroid after clinical presentation of non-productive cough, shortness of breath, and fever.

**Table 2:** Literature search: case reports

Study	Trastuzumab dosing regimen	History of smoking/lung disease	Symptom onset	Clinical presentation	Treatment used	Resolution of symptoms after treatment
Alkan, 2019 [17]	Trastuzumab emtansine 3.6 mg/kg every 3 weeks	No	3 days after the second cycle	Dyspnea and non-productive cough	1 mg/kg methylprednisolone, inhaler salbutamol, moxifloxacin, and supportive care	After one week of therapy, dyspnea gradually disappeared and radiological findings improved
Vahid and Mehrotra, 2006 [5]	Trastuzumab infusions biweekly	No	A few hours after the 6th cycle	Shortness of breath	Methylprednisolone 45 mg IV daily	Symptoms resolved and marked improvement of left lung infiltrates was seen after 3 weeks
Costa et al., 2017 [18]	Trastuzumab 8 mg/kg loading dose followed by 6 mg/kg every 21 days	No	14th day of the 5th cycle	Shortness of breath on exertion with associated dry cough	Prednisone 40 mg by mouth daily	Yes, but then symptoms reoccurred after 2 more cycles of trastuzumab
Sugaya et al., 2017 [12]	Patient 1: triweekly trastuzumab; patient 2: one administration of trastuzumab; patient 3: triweekly trastuzumab	Patient 1: no; patient 2: no; patient 3: no	Patient 1: 4th day of the 5th course; patient 2: 3 hours after the first administration of trastuzumab; patient 3: symptoms started at the start of the 3rd course out of the 4 courses of chemotherapy but was hospitalized on the 6th day of the first course of monotherapy	Patient 1: low-grade fever and cough; patient 2: low-grade fever and right lung-dominant coarse crackles; patient 3: transient flu-like symptoms and fine crackles bilaterally	Patient 1: steroid semi-pulse therapy with concurrent administration of meropenem and sulfamethoxazole/trimethoprim; patients 2 and 3: prednisolone 30 mg orally daily	Patients 1 and 2: symptoms subsided within 7 days of the treatment; patient 3: symptoms resolved, and serum levels of KL-6 decreased gradually over 8 months
Pepels et al., 2008 [19]	Triweekly administered trastuzumab	No	Patient was treated for 5 months with a combination therapy of docetaxal/trastuzumab, which was then discontinued and trastuzumab was used as monotherapy. Onset occurred 8 days after the first administration of trastuzumab monotherapy. Onset occurred again 3 weeks after previous hospitalization when trastuzumab was reinitiated.	First event: dry cough and progressive dyspnea; second event: fever, progressive dyspnea, and wheezing	First event: amoxicillin with clavulanic acid and levofloxacin; second event: prednisolone 40 mg once daily	Symptoms resolved at both events.
Ye et al., 2019 [20]	Information not available	Information not available	Onset occurred two months after initiation of maintenance therapy	Minimally productive cough and low-grade fever	IV corticosteroids were administered	Symptoms resolved within 48 hours

**Table 3:** Literature search: clinical trials

Study name	Study design	Sample size	Cases of interstitial pneumonitis	Trastuzumab dosing regimen	Patient outcome
Modi et al., 2020 [9]	Two-part, open-label, single-group, multicenter, phase 2 study	253 patients	8 patients	Trastuzumab deruxtecan 6.4 mg/kg dose every 21 days	Five of the patients recovered and three died
Romond et al., 2005 [11]	Randomized phase III clinical trials	Trial B-31: 2,043 patients; trial N9831: 1,633 patients	Trial B-31: 4 patients; trial N9831: 5 patients	Trial B-31: trastuzumab 2mg/kg dose every week; trial N9831: trastuzumab 2 mg/kg dose every week	Trial B-31: three of the patients recovered and one died; trial N9831: four of the patients recovered and one died
Modi et al., 2020 [10]	First-in-human, phase I, nonrandomized, open-label, multiple-dose study	54 patients	8 patients	Trastuzumab deruxtecan 6.4 mg/kg dose every 21 days	Five of the patients recovered and three died
Tsurutani et al., 2020 [16]	Dose-expansion, phase I study	60 patients	5 patients	Trastuzumab deruxtecan 6.4 mg/kg dose every 21 days	Four of the patients recovered and one died

This patient presented with shortness of breath and a worsening dry cough that she experienced for the past three weeks after their second maintenance cycle of trastuzumab. COVID-19, pulmonary embolism, infection, pulmonary edema, and pneumonia were all ruled out before the diagnosis was made for trastuzumab-induced interstitial pneumonitis. Within 24 hours of presenting to the hospital, the patient was started on methylprednisolone for four days. The patient was then transitioned to prednisone, and by day 5, the patient had responded well enough to treatment to be discharged. The Naranjo algorithm was used to assess for a causal relationship between trastuzumab and interstitial pneumonitis. This patient had a score of 7 (probable adverse drug reaction).

## Conclusions

Drug-induced interstitial pneumonitis has been reported in patients receiving HER2 therapies, cytotoxic agents, and non-cytotoxic agents, but the limited provider exposure to this adverse effect makes monitoring and diagnosis difficult. In summary, with the increased use of trastuzumab treatment, physicians should be aware of this rare, but potentially harmful adverse reaction.
